# A Surgical Model of Permanent and Transient Middle Cerebral Artery Stroke in the Sheep

**DOI:** 10.1371/journal.pone.0042157

**Published:** 2012-07-27

**Authors:** Adam J. Wells, Robert Vink, Peter C. Blumbergs, Brian P. Brophy, Stephen C. Helps, Steven J. Knox, Renée J. Turner

**Affiliations:** 1 Adelaide Centre for Neuroscience Research, School of Medical Sciences, University of Adelaide, Adelaide, South Australia, Australia; 2 Tissue Pathology, South Australia Pathology, Adelaide, South Australia, Australia; 3 Department of Neurosurgery, Royal Adelaide Hospital, Adelaide, South Australia, Australia; 4 Department of Radiology, Royal Adelaide Hospital, Adelaide, South Australia, Australia; Julius-Maximilians-Universität Würzburg, Germany

## Abstract

**Background:**

Animal models are essential to study the pathophysiological changes associated with focal occlusive stroke and to investigate novel therapies. Currently used rodent models have yielded little clinical success, however large animal models may provide a more suitable alternative to improve clinical translation. We sought to develop a model of acute proximal middle cerebral artery (MCA) ischemic stroke in sheep, including both permanent occlusion and transient occlusion with reperfusion.

**Materials and Methods:**

18 adult male and female Merino sheep were randomly allocated to one of three groups (n = 6/gp): 1) sham surgery; 2) permanent proximal MCA occlusion (MCAO); or 3) temporary MCAO with aneurysm clip. All animals had invasive arterial blood pressure, intracranial pressure and brain tissue oxygen monitoring. At 4 h following vessel occlusion or sham surgery animals were killed by perfusion fixation. Brains were processed for histopathological examination and infarct area determination. 6 further animals were randomized to either permanent (n = 3) or temporary MCAO (n = 3) and then had magnetic resonance imaging (MRI) at 4 h after MCAO.

**Results:**

Evidence of ischemic injury in an MCA distribution was seen in all stroke animals. The ischemic lesion area was significantly larger after permanent (28.8%) compared with temporary MCAO (14.6%). Sham animals demonstrated no evidence of ischemic injury. There was a significant reduction in brain tissue oxygen partial pressure after permanent vessel occlusion between 30 and 210 mins after MCAO. MRI at 4 h demonstrated complete proximal MCA occlusion in the permanent MCAO animals with a diffusion deficit involving the whole right MCA territory, whereas temporary MCAO animals demonstrated MRA evidence of flow within the right MCA and smaller predominantly cortical diffusion deficits.

**Conclusions:**

Proximal MCAO can be achieved in an ovine model of stroke via a surgical approach. Permanent occlusion creates larger infarct volumes, however aneurysm clip application allows for reperfusion.

## Introduction

The current clinical treatment of acute occlusive middle cerebral artery (MCA) stroke is directed towards early reperfusion of the ischemic brain in a timely fashion by thrombolysis or thrombectomy [1], [2]. However, the majority of MCA occlusion (MCAO) stroke patients either do not meet the strict criteria or fail to receive significant reperfusion, and are managed with best medical therapy and decompressive craniectomy for the malignant MCAO variant [3], [4], [5]. Over 1000 agents have shown to be efficacious in preclinical evaluation, with only tissue plasminogen activator translating into a successful clinical therapy [6], [7], [8]. This poor translation has led many researchers to believe that rodent models have limited predictive value and that alternate large animal models are likely to become important in future translational research [9], [10]. Given that the number of individuals that will suffer a stroke in the next decade will rapidly increase [11], novel therapies that can limit or reverse ischemic injury at a cellular level are urgently required.

An ovine model of permanent focal cerebral ischemia has recently been developed to capitalize on the advantages of large animal translation [12], however this model offers only permanent vessel occlusion and the technique of MCA exposure and occlusion was only briefly described. Accordingly, the aims of our study were to develop a sheep model of surgical proximal MCA exposure and occlusion, to establish the viability of different methods of vessel occlusion including those facilitating reperfusion, and to determine the extent and pattern of ischemic injury with different methods of occlusion with or without reperfusion. We describe in detail a technique we have developed of focal MCA occlusion in the sheep that allows investigation of either permanent or temporary proximal vessel occlusion.

## Materials and Methods

### 2.1 Experimental Procedure

All experimental protocols were approved by the Animal Ethics Committees of the University of Adelaide and SA Pathology, and conducted according to guidelines established for the use of animals in experimental research as outlined by the Australian National Health and Medical Research Council code of practice for the care and use of animals for scientific purposes (7^th^ edition, 2004).

### 2.2 Animals and Experimental Design

18 adult male and female Merino sheep 18–24 months old (mean weight 50.1+/−5.8 kg) were allocated to the study. Animals were intra-operatively randomized to permanent occlusion (n = 6), 2 h temporary vessel occlusion followed by reperfusion (n = 6) or sham surgery (n = 6). Anesthesia was induced with intravenous thiopentone (1000 mg in 20 mL, Jurox Pty Ltd, Australia) and maintained with 1.5% inhalational isoflurane (Veterinary Companies of Australia Pty Ltd, Australia) in a mixture of oxygen and room air, plus intravenous ketamine (Parnell Australia Pty Ltd, Australia) infusion 4.0 mg/kg/hr via a femoral venous line. These two agents were used to avoid the intrinsic neuroprotective properties of either above certain doses, but to maintain a twilight general anesthesia. With the animal supine, an arterial catheter was placed in the right femoral artery for continuous blood pressure monitoring and periodic arterial blood gas sampling. The animal was then placed prone in the sphinx position, burr holes were placed symmetrically in left and right parietal bones posterior to the coronal suture and approximately 20 mm from the sagittal suture, dura was perforated and skull bolts secured. In the left bolt, a Codman microsensor intracranial pressure (ICP) probe (Codman & Shurtleff Inc., MA) was calibrated and inserted intraparenchymally to a depth of approximately 15 mm, and in the right a LICOX brain tissue oxygen (PbtO_2_) probe (Integra LifeSciences, NJ) was inserted and secured to a depth of approximately 10 mm, such that its oxygen sensing tip was positioned in the parietal lobe cortex supplied by the right MCA.

### 2.3 Surgical Approach to the MCA

**Figure 1:**
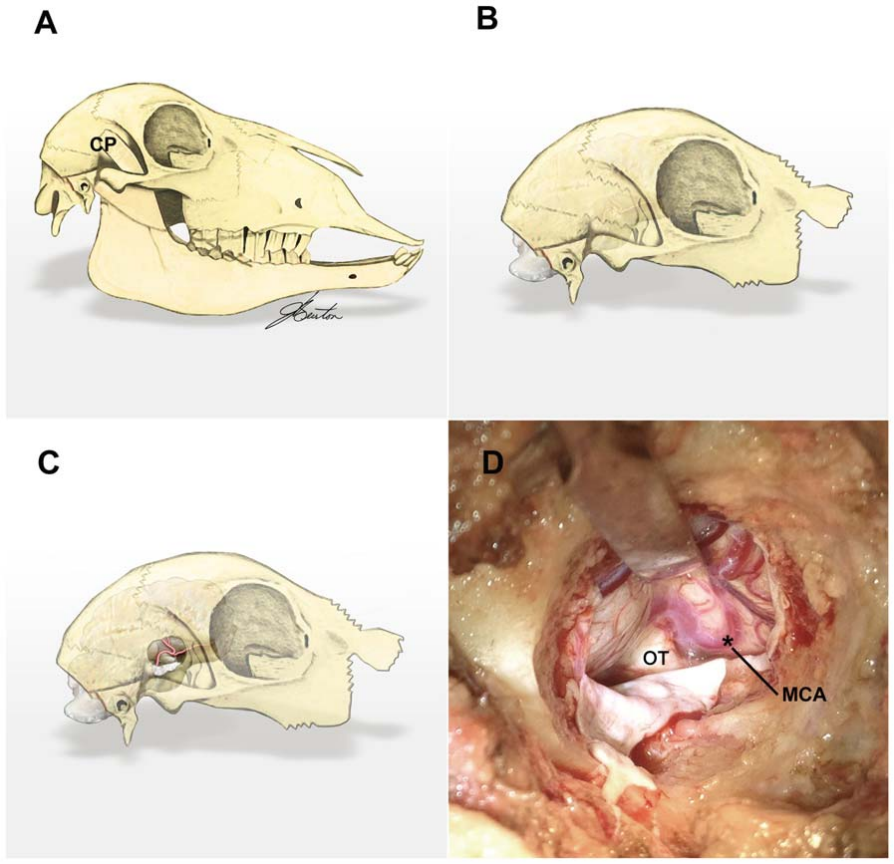
Surgical approach to the proximal MCA. The coronoid process of the mandible (A) is fractured near its base and removed to expose the skull overlying the MCA (B). A small craniotomy and durotomy reveals the proximal part of the vessel (C). Intraoperative photography demonstrating the proximal MCA looping at the skull base (D); asterisk marks the point of occlusion. CP, coronoid process; MCA, middle cerebral artery; OT, optic tract.

With the animal still in the sphinx position, the head was tilted to the left ninety degrees and secured on a support to facilitate a right MCA approach, utilizing gravity to lift the cerebrum away from the bony skull base during intracranial surgery. Wool between the eye and ear was shorn and a 50 mm vertical incision made terminating at the zygomatic arch. Temporalis and other muscles of mastication were divided and stripped from the coronoid process of the mandible. Removing the coronoid process is necessary to perform a craniotomy adequate to access the proximal MCA, and it was thus fractured at its base level with the zygomatic arch after muscle stripping (Figure 1A–B). The remaining masticators were then divided and stripped from the outer table of the cranium with monopolar diathermy (Covidien, Ireland) as far forward as the fibrous ring attaching the posterior orbit to the concave border of the parietal bone. A small craniotomy was performed over the junction of the parietal and squamous temporal bones with a high-speed pneumatic drill using a 5 mm cutting burr (Midas Rex, Medtronic, MN), taking care not to breach the dura beneath. Bone was carefully removed anteroinferiorly with a Kerrison rongeur from the greater wing of sphenoid to aid in later exposure of the proximal MCA at the circle of Willis. Our goal was for the inferior margin of the craniotomy to run approximately level with floor of the middle cranial fossa, with the concave dural surface marking the junction of frontal lobe with temporal lobe in the center of the craniotomy (Figure 1C). The anterior component of the craniotomy was frequently the most difficult part of the exposure but also the most critical in order to achieve satisfactory proximal MCA access. An adequate amount of bone removal over the squamous temporal was necessary to aid safe brain retraction and manipulation to access the terminal ICA.

A horseshoe shaped durotomy was performed with an inferiorly based flap. With a well-placed craniotomy, the cortical branches of MCA and its bifurcation point were seen running in the subarachnoid space on the surface of the brain. All intradural work was carried out with loupe magnification and a head mounted light source (Surgical Acuity, WI). Careful suction of cerebrospinal fluid (CSF) from the arachnoid cisterns of the anterior circulation promotes brain relaxation, and increases visibility in addition to minimizing retraction injury. The cortical branches were followed proximally, and with gentle upwards retraction of the anterior temporal lobe and careful aspiration of CSF from the prechiasmatic cistern, the terminal internal carotid artery (ICA) was identified looping around the optic tract medial to where the free tentorial edge meets the roof of the cavernous sinus (Figure 1D). Refer to online (Video S1) for a multimedia file demonstrating the surgical approach to the proximal MCA, including placement of a temporary aneurysm clip.

#### 2.3.1 Neurovascular Anatomy

**Figure 2:**
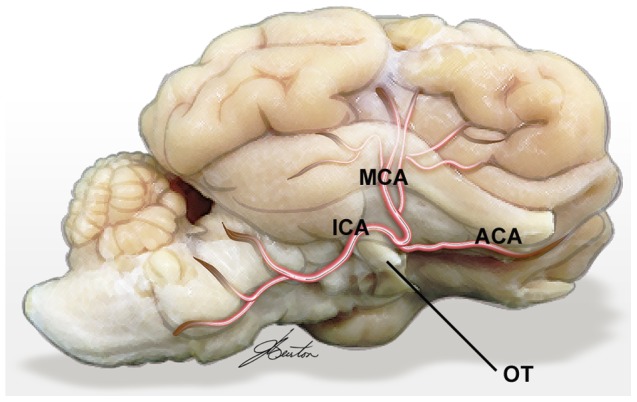
Neurovascular anatomy of the sheep, anterior circulation, inferolateral view, right side. ICA sweeps around the optic tract before bifurcating terminally into MCA and ACA. MCA loops backwards before running laterally on the surface of the brain and dividing into terminal branches. ACA, anterior cerebral artery; ICA, internal carotid artery; MCA, middle cerebral artery;. OT, optic tract.

The sheep neurovascular anatomy has been reported previously [13], [14], however for the purposes of this model a brief description of the relevant surgical anatomy is described and illustrated. As with most ruminants, the sheep has an extradural rete mirabile at the skull base from which the intradural ICA arises to provide blood supply to the majority of the supratentorial structures. Similar to the human, the terminal intradural ICA bifurcates to the anterior cerebral artery (ACA) and MCA, however in the human the intradural ICA course is short whereas in the sheep the it runs anteriorly for a short distance, sweeping forwards from its dural origin lateral to the optic tract adjacent to the optic chiasm and inferior to the large olfactory lobe, where the ACA and MCA originate (Figure 2). The MCA runs forwards to approach the optic nerve but then turns back on itself posterolaterally in its proximal segment on the undersurface of the posterior frontal lobe. It turns again to run up laterally on the cortical surface and bifurcates early, just anterior to the inferior pole of the temporal lobe, into anterior and posterior trunks. These trunks are often visible for quite a distance on the lateral cortical surface at the junction of frontal and temporal lobes, before separating and dividing further into terminal cortical branches. Unlike the human, the sylvian fissure in the sheep is shallow and does not require dissecting to access the MCA along its course from origin to its terminal branches.

#### 2.3.2 MCA Occlusion

After exposure of the proximal MCA, animals were randomized into one of three groups as follows: 1) sham, in which the proximal MCA was dissected but not occluded; 2) permanent occlusion, in which the proximal MCA was occluded via Malis bipolar diathermy forceps (Valleylab Inc., CO); and 3) temporary occlusion with reperfusion, in which the proximal MCA was occluded with application of a Sugita temporary mini straight aneurysm clip (Mizuho Medical Inc, Japan) which was removed 2 h later. The exposed brain was irrigated with saline during surgery to prevent drying out of the cerebral cortex, particularly for temporary occlusion animals in which there was a 2 h delay between aneurysm clip application and wound closure. The site of vessel occlusion was standardized to the proximal MCA within 3 mm of its origin, as depicted by the asterisk in Figure 1D.

After sham surgery, permanent MCA occlusion or aneurysm clip release, the dura was approximated and closed watertight with ethyl cyanoacrylate (Bostik, Australia) and reinforced with dental acrylic cement (Lang Dental, IL) which was manipulated into the edges of the craniotomy, maintaining the shape of the cranial cavity and homeostasis of intracranial pressure dynamics. The wound was closed in layers and the head was then returned to a neutral position for monitoring under anesthesia.

### 2.4 Histological Examination

At 4 h following the onset of vessel occlusion or sham surgery, animals were administered intravenous heparin (5000 I.U./5 ml; Pfizer, NY) and killed via common carotid perfusion fixation with 10% neutral-buffered formalin [15]. The head was stored overnight at 4°C to minimize artifact from premature tissue manipulation [16]. Brains were removed and stored in formalin for a minimum of 7 d prior to being processed, embedded in paraffin wax and sectioned coronally at 5 mm intervals for histological examination by H&E, albumin immunohistochemistry (dilution 1∶20000, ICN Pharmaceuticals Australasia Pty Ltd, Australia), and Weil's stain. Infarct area was determined by calculating the percentage of infarct volume on H&E in a whole coronal section at a level through the optic chiasm, encompassing head of caudate, anterior thalamus, putamen, internal capsule and the area of greatest cortical MCA supply. Edema was corrected for by a modified Swanson calculation [17].

### 2.5 Magnetic Resonance Imaging

A further 6 animals (mean weight 55.8+/−5.2 kg) were randomized to permanent MCAO (n = 3) or 2 h temporary MCAO followed by reperfusion (n = 3) via an identical protocol outlined above but without insertion of ICP or PbtO_2_ monitors. At 4 h after vessel occlusion animals were placed in a 1.5T Siemens Sonata (Siemens AG, Munich, Germany) Magnetic Resonance Image (MRI) machine for a sequence protocol that included magnetic resonance angiography (MRA), diffusion weighted imaging (DWI), fluid attenuated inversion recovery (FLAIR), and T1 and T2 weighted images.

### 2.6 Statistical Analysis

All data is expressed as mean +/− standard deviation. Physiological data (arterial blood pressure, ICP, PbtO_2_, PO_2_, PCO_2_) was analyzed using two-way analysis of variance (ANOVA) followed by individual Bonferroni tests (Prism Version 5.0 d, Graphpad, CA). Physiological parameters were analyzed pre-MCAO or sham surgery, and at 30 minute intervals until the completion of the experiment. Lesion volume data was analyzed by individual student t-test. A p-value of p<0.05 was considered significant.

## Results

### 3.1 Surgery

All experimental procedures were carried out without complication. There were no on-table mortalities or unexpected events. The mean time from the beginning of the operation to vessel occlusion or sham surgery was 76.1+/−18.7 mins (range 53 to 105 mins).

### 3.2 Physiological Parameters

**Figure 3:**
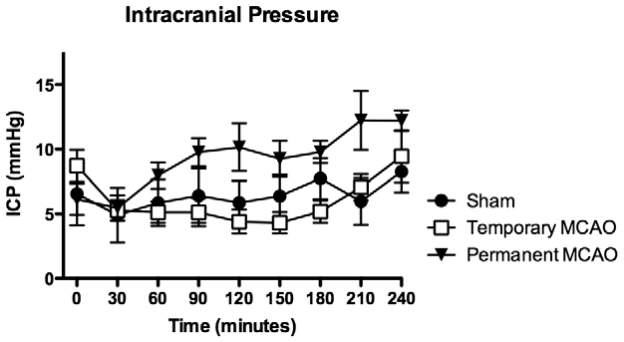
ICP after surgical MCAO, first 4 hours. ICP falls initially in all three groups due to dural opening and CSF aspiration. After skull reconstruction, ICP slowly rises above pre-craniotomy levels after permanent MCAO, but without any significant difference between groups.

**Figure 4:**
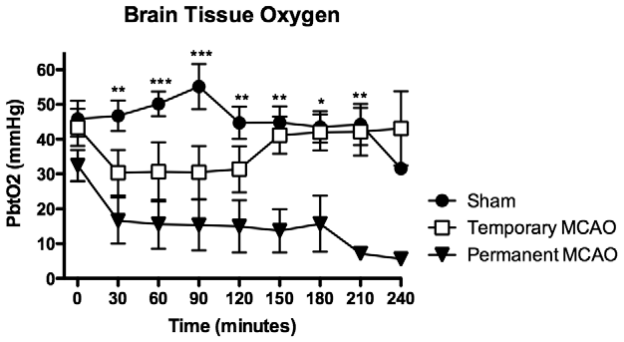
PbtO_2_ after surgical MCAO, first 4 hours. After permanent MCAO, PbtO_2_ falls significantly compared to sham. PbtO_2_ begins to rise again upon release of the temporary aneurysm clip in the transient occlusion group, however remains low in the permanent occlusion group. Sham animals demonstrate no significant change for the duration of the monitoring period. *, p<0.05; **, p<0.01; ***, p<0.001 (permanent MCAO compared to sham).

Basic physiological parameters are expressed in table 1. For all groups, there was no statistically significant difference for mean arterial PO_2_, PCO_2_, or blood pressure at any of the recorded time intervals. Mean ICP was 7.1+/−1.4 mmHg prior to craniotomy, falling to 5.2+/−0.3 mmHg upon opening the dura and rising to 10.0+/−2.0 mmHg by the end of the 4 hour monitoring period, with no significant difference between groups (Figure 3). Mean PbtO_2_ was 40.6+/−7.2 mmHg prior to vessel occlusion or sham. PbtO_2_ remained stable throughout the monitoring period in sham animals, however decreased in both MCAO groups for minimum values of 5.6+/−1.0 mmHg in permanent occlusion animals at 240 mins and 30.5+/−17.0 mmHg in transient occlusion animals at 90 mins (Figure 4). For almost the entire duration of the monitoring period, PbtO_2_ after permanent MCAO remained significantly reduced (30–210 mins), however after temporary MCAO PbtO_2_ returned to and remained at baseline levels upon release of the aneurysm clip after a period of non-significant reduction during clip application (41.2+/−11.9 mmHg at 150 mins vs 44.8+/−11.4 mmHg for sham).

**Table 1:** Physiological data.

Group	PO_2_ (mmHg) +/− SD	PCO_2_ (mmHg) +/− SD	ABP (mmHg) +/− SD
Sham	112.1+/−15.7	37.0+/−5.0	104.5+/−13.5
Permanent MCAO	101.4+/−25.8	41.2+/−8.6	94.7+/−7.6
Temporary MCAO	115.2+/−20.5	39.4+/−8.6	101.3+/−2.9

Mean blood gas and arterial blood pressure measurements for all time intervals by group. MCAO, middle cerebral artery occlusion; PO_2_, arterial partial pressure of oxygen; PCO_2_, arterial partial pressure of carbon dioxide; ABP, mean arterial blood pressure.

### 3.3 Post mortem and gross pathological changes

Macroscopic changes consistent with acute cerebral ischemia were demonstrated in all permanent and temporary vessel occlusion animals. Sham animals demonstrated evidence of surgery in the region of the right proximal MCA, however the vessel was patent, the brain well perfused in all territories and macroscopically showed no evidence of injury. Permanent occlusion animals showed softening of the MCA supplied cortex adjacent to the firm formaldehyde perfuse-fixed brain of the anterior and posterior cerebral artery territories, whereas temporary occlusion and sham animals had uniformly perfuse-fixed brains. On coronal sections, permanent occlusion brains were swollen in the MCA territory with ipsilateral compression of the lateral ventricle and midline shift towards the contralateral side. Temporary occlusion animals demonstrated subpial petechial hemorrhages but a lesser degree of swelling. The proximal pre-bifurcation MCA was confirmed as the vessel occluded in both MCAO groups at post mortem.

### 3.4 Histopathology and infarct area

**Figure 5:**
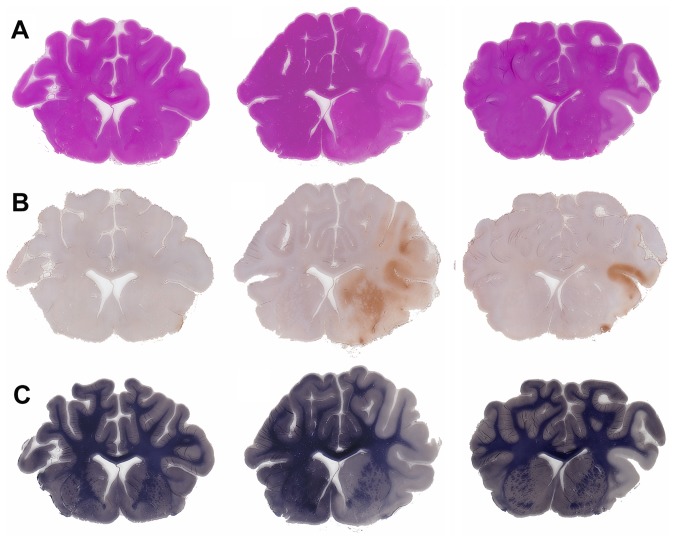
Histopathology. (A) H&E stain for sham (left), permanent MCAO (center), temporary MCAO (right). (B) Albumin immunostain for sham (left), permanent MCAO (centre), temporary MCAO (right). Weil stain for sham (left), permanent MCAO (centre), temporary MCAO (right). MCAO, middle cerebral artery occlusion.

**Figure 6:**
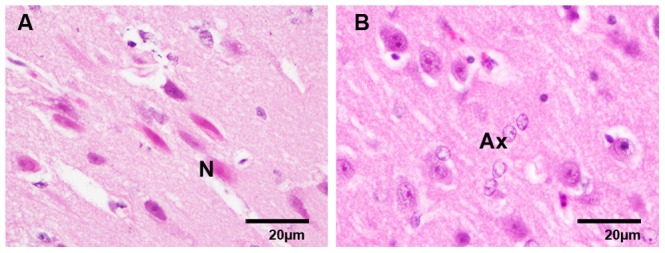
Histopathology. ‘Red neurons’ (A) and astrocytic nuclear swelling (B). H&E ×400. N, neuron; Ax, axon.

No microscopic evidence of ischemic injury was observed in sham animals. Conversely, MCAO animals demonstrated signs of ischemic injury within the MCA territory (cortical and subcortical structures), as evidenced by areas of pallor on H&E staining correlating with albumin extravasation and pallor on Weil staining (Figure 5). The H&E changes seen were due to microvacuolation of the neuropil which varied in different areas from fine to coarse microvacuolation. The cortical neurons in the coarsely vacuolated areas showed a spectrum of change varying from marked cytoplasmic shrinkage and pyknotic nuclei to early eosinophilia of the shrunken cytoplasm (‘red neurons’ corresponding with acute ischemic change, Figure 6A). Vesicular swelling of astrocytic nuclei was present before neuronal changes were evident at 4 h survival (Figure 6B).

**Figure 7:**
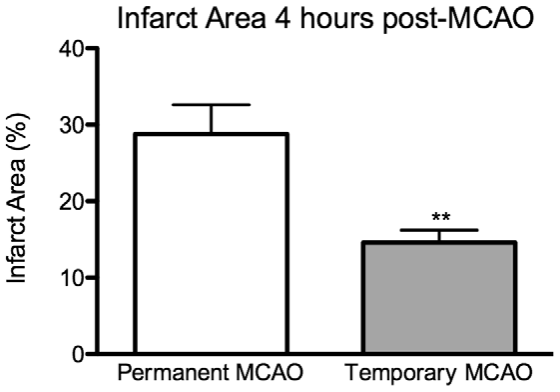
Infarct area at 4 hours. Infarct areas expressed as percentage of ischemic brain tissue identified on H&E staining on coronal section level with the optic chiasm; areas have been corrected for edema. **, p<0.01.

The ischemic area was significantly larger in permanent occlusion animals than temporary occlusion (Figure 7, 28.8% vs 14.6%, p<0.01); in permanent MCAO there was evidence of ischemia involving the majority of the MCA territory, as well as subcortical structures (caudate nucleus, putamen), whereas ischemic areas in temporary MCAO animals were typically patchy in the MCA supplied cortex and with variable involvement of sub-cortical structures (Figure 5). A small amount of albumin extravasation was visible at the site of the craniotomy in sham animals suggesting not-insignificant injury to the underlying cortex secondary to the surgical approach and/or closure.

### 3.5 Magnetic Resonance Imaging

**Figure 8:**
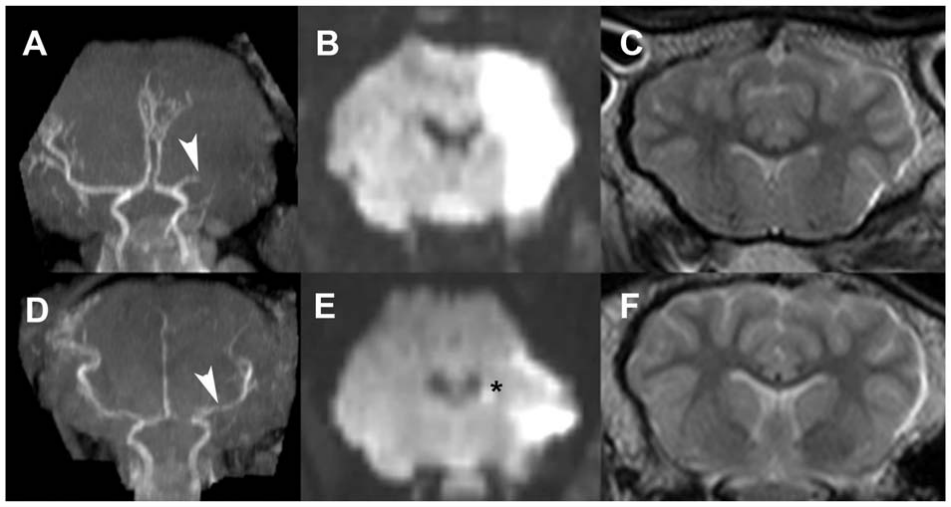
MRI at 4 hours after MCAO, coronal orientation. Permanent MCAO MRA (A), DWI (B) and T2 weighted imaging (C). Temporary MCAO with reperfusion MRA (D), DWI (E) and T2 weighted imaging (F). Arrowhead on MRA indicates site of arterial occlusion. Asterisk indicates restricted diffusion at caudate head after temporary MCAO. DWI, diffusion weighted imaging; MCAO, middle cerebral artery occlusion; MRA, magnetic resonance angiogram; MRI, magnetic resonance imaging.

Permanent MCAO animals were characterized by complete proximal MCA occlusion on MRA with poor collateralization (Figure 8A), and restricted diffusion through the entire right MCA territory including the majority of the basal ganglia (high DWI signal in Figure 8B). There was increased signal at the site of craniotomy on T2 weighted imaging consistent with surgical manipulation but no FLAIR signal abnormalities identified, no blood products visualized and no evidence of mass effect (Figure 8C).

Temporary MCAO animals demonstrated a focal deficit on MRA at the proximal MCA consistent with clip occlusion but with reconstituted flow to the distal MCA branches (normal distal MCA filling, Figure 8D). DWI showed restricted diffusion at the right caudate head and genu of the internal capsule in addition to areas of restricted cortical diffusion (Figure 8E). As per permanent MCAO, there was T2 evidence of surgical manipulation but no FLAIR signal abnormalities and no blood products identified (Figure 8F).

## Discussion

Animal models remain the most widely used approach to study the pathogenesis of ischemic stroke and determine the efficacy of various drug therapies. Large animal models of experimental stroke were common until the mid 1980s when the intraluminal thread model of MCAO was introduced in the rat [18], and although the rodent model offers many advantages, such as low cost, known physiological database and the ability to investigate knock out genes [19], the biological interspecies differences and poor clinical translation suggests that an animal model that is more clinically relevant is necessary [9], [20], [21]. Despite the inherent disadvantages of working with large animals [21], they are potentially better suited to investigate stroke pathology and study treatment efficacy [10]. There are a number of non-primate large animals models of acute ischemic stroke in the current literature, each with their own inherent advantages and disadvantages. Rabbits have an arterial anatomy that permits endovascular autologous clot occlusion of various intracranial arteries including the MCA [22] and the basilar artery [23], however their brains are lissencephalic like the rat and not generally representative of the human gyrencephalic brain. Cats are of a similar size to rabbits and have a gyrencephalic brain, however their vascular anatomy precludes an endovascular approach to occlusion and the surgical approach is transorbital [24]. Both rabbits and cats are considerably smaller than sheep or pigs, and although weight itself does not predict phylogenetic order, larger brains allow for easier surgical manipulation and limit the requirement of higher resolution imaging. Boltze et al. have demonstrated permanent extrinsic MCAO in a sheep model, however their site of surgical occlusion targets distal cortical branches and spares the proximal trunk and deep perforators [12]; it also does not allow for reperfusion. Watanabe et al. similarly produced permanent MCAO in a pig model in which the vessel was occluded proximally, however this required a transorbital approach and subsequent persistent craniotomy defect [25]. Non-human primates (NHP) remain the closest to humans phylogenetically, however endovascular autologous thromboembolic NHP models produce unreliable anterior circulation stroke patterns and their use worldwide is increasingly limited by restricted ethics approval [10], [26].

One reason why NHP are so attractive for translational stroke research is their remarkable anatomical similarity to the human brain, such that acute proximal MCAO results in basal ganglia and white matter infarction in addition to cortical stroke [26]. Considering the importance of anatomical similarity, the sheep brain appears highly promising as a substitute for human stroke research. Key features are its gyrencephalic pattern, dense white matter tracts, strong fibrous dura mater and tentorium cerebelli, and large size compared with the rodent, rabbit or cat brain. The neurovascular anatomy is very similar to the human [10] with the exception of an extradural rete mirabile [27], which precludes an endovascular or embolic approach to MCAO. In addition to a large area of cerebral cortex, the human MCA may give rise to perforating lenticulostriate arteries which take origin from the first two MCA segments, the majority arising from the prebifurcation M1 segment with a smaller number from the postbifurcation M1 and less commonly M2 segments, but may also arise from the ACA or ICA [28]. Human lenticulostriates supply the head and body of the caudate nucleus, the superior part of the internal capsule and the lateral part of the globus pallidus after traveling through the anterior perforated substance. The surface of the well developed rhinencephalon in the sheep corresponds to the anterior perforated substance [29]; perforating arteries in this region are derived from the ACA and MCA, either as single trunks from parent vessels or as trunks divided into cortical branches of the olfactory lobe. These perforators have many anastomoses creating conditions for a rich collateral supply [29], with the exception of arterioles distributed to the amygdaloid nucleus [30], [31]. To include the deep perforating branches when performing an occlusive MCA stroke it is important to expose the artery proximally at its origin at the terminal ICA bifurcation.

Transient ischemia in addition to permanent vessel occlusion is a major advantage of this study and has the ability to significantly improve upon the existing ovine model [12]. Furthermore, we have demonstrated significantly different lesion areas between transient and permanent proximal MCAO. Temporary vessel occlusion with reperfusion strengthens the translational potential, and recent STAIR recommendations have suggested that preclinical testing of neuroprotective agents should be conducted in multiple species and with both temporary and permanent occlusion models [32]. MCAO by means of an aneurysm clip provides further advantages. The clip applicators are ergonomically easy to handle, are adept at placing clips within small surgical exposures under the guidance of illuminated magnification, and temporary clips allow for repositioning with little risk of significant endothelial damage [33] if initial placement is deemed inadequate. In addition, aneurysm clips can be placed essentially atraumatically after freeing the MCA from its overlying arachnoid. The clip may be left on the vessel for any length of time before reperfusion, or alternatively could be left on permanently. For permanent vessel occlusion we used bipolar electrocautery, which we found easier to consistently occlude the MCA at its most proximal part with less bony dissection and brain retraction and little or no arachnoid dissection. It does, however, create a discrete area of focal electrocautery trauma to the adjacent brain tissue. Parenchymal injury may be limited by using a low current setting and restricting cauterization to only a short segment of the proximal MCA under direct visualization. An aneurysm clip left on permanently would avoid electrical trauma but at the cost of increased surgical difficulty.

### PbtO_2_ and Cerebral Blood Flow

The use of a LICOX system to measure PbtO_2_ has advantages and disadvantages, and although it is not a true surrogate for cerebral blood flow (CBF), it may correlate well with regional CBF, particularly after brain injury [34]. In the context of this model, in which we measured the partial pressure of oxygen within a focal area of brain tissue at the tip of the probe, PbtO_2_ may be seen as a reasonable substitute for measuring CBF. Cerebral arteries are classically thought of as end-arteries, therefore detection of brain tissue hypoxia within one vascular zone should be representative of all cerebral structures supplied by that artery. However considerable inter-individual differences in collateral supply means this is not always the case [35]. Furthermore, problems with consistent placement of the probe tip within the same vascular zone could influence the PbtO_2_ results, such that if the LICOX probe tip lay within a watershed zone partially supplied by posterior or anterior cerebral arteries, or even placed too deep to be representative of a cortical MCA zone. In such circumstances, PbtO_2_ levels may have remained falsely elevated when in fact the ischemic core was grossly hypoxic. Indeed, this was observed in 2 temporary MCAO animals, in which there was no reduction of PbtO_2_ despite demonstrable ischemic change on H&E, and contributed to the wide standard deviations and lack of significance in this group. PbtO_2_ levels in permanent MCAO animals were significantly reduced, reaching a minimum level of 5.6 mmHg by 240 mins after MCAO. Normal values for PbtO_2_ in the human are 33.0–47.9 mmHg [36], and in the sheep 44.0–52.0 mmHg [37], which compares favorably with our mean baseline measurement of 40.6 mmHg. In animal models and clinical data of traumatic brain injury there is evidence that neurological outcomes are poorer the longer PbtO_2_ is <15 mmHg, and in permanent MCAO animals this occurred within 120 mins and persisted for the remainder of the experimental period, however did not fall below 30 mmHg in the temporary MCAO group. There was a noticeable drop in PbtO_2_ levels in sham animals at 240 mins, which can be explained by missing data at this time point in 4 of the 6 animals in this group; this also contributed to the lack of statistical significance between sham and permanent MCAO at 240 mins. Despite its disadvantages, the LICOX system is easy to use and minimally invasive, provides rapid, continuous and accurate measurements [36], is freely commercially available and is widely reported in the literature with regard to both human and animal outcomes, particularly in relation to traumatic brain injury [37], [38].

### Surgical approach and ICP

Previous large animal models have frequently utilized a transorbital approach to the MCA in order to minimize brain retraction [39]. We ultimately elected to approach the MCA transcranially for several reasons. Firstly to avoid enucleation; in anticipation of evolving this method into a survival MCAO stroke model, we felt that clinical observation of both eyes and visual fields would be important. Secondly, a well-placed transcranial approach gives direct access to the proximal MCA, from its origin to beyond its cortical bifurcation, providing the option of performing vessel occlusion at the main trunk or at one or more distal branches. Finally our approach allows for watertight dural closure with replacement of the removed bone with a suitable substitute, which we found to be important for ICP dynamics. A watertight dural closure can be achieved with cyanoacrylate alone, which we observed to not produce any significant heat during polymerization, however the ethyl monomer is recognized to produce focal cerebral injury [40] which may explain the small amount of albumin uptake on immunostaining of sham animals. This could potentially be eliminated by using the less traumatic isobutyl cyanoacrylate monomer, or a commercial albeit more expensive dural closure product such as DuraSeal (Confluent Surgical Inc., MA). Secondary brain injury such as raised ICP can contribute significantly to morbidity and mortality associated with large strokes [41], so maintaining the integrity of the closed cranial cavity after vessel occlusion is an important component of this model. ICP decreases on opening the dura and aspirating CSF, however rises again to pre-craniotomy levels towards the end of the 4-hour monitoring period after dural and bone reconstruction and as CSF reaccumulates. Our results demonstrated a relatively low early mean ICP after temporary clip occlusion, which may in part be explained by the craniotomy remaining open for 2 h longer in this group than the other two groups. This delayed closure is necessary to remove the aneurysm clip, however postpones reconstitution of ICP homeostasis after craniotomy and needs to be taken into consideration. The supratentorial space can generally be considered a uniform compartment with regards to ICP, however ICP gradient development after unilateral stroke remains controversial. In one model of NHP stroke, significant gradients were observed in animals with >20% infarct volume [42]. Another report in malignant human stroke demonstrated equal bilateral ICP changes for the majority of the monitoring period, only developing a gradient after the development of transtentorial herniation and brainstem compression [43]. Clearly having the PbtO_2_ sensor placed within the ischemic tissue was important, hence the LICOX monitor was placed ipsilaterally, and although the sheep brain is considerably larger than the rodent and permits insertion of multiple probes, having the current setup versus two ipsilateral probes was favorable to limit overcrowding in the surgical field. Considering the area of ischemic tissue at 4 h in the permanent MCAO group, we predict to observe a significant ICP rise in longer periods of monitoring as occurs in human malignant MCA stroke [44], and this has been identified as an area of future study to help confirm the translational importance of this model.

### MRI

MRI adds significantly to the translational potential of our model. Firstly, we have demonstrated with MRA that permanent MCAO results in complete and sustained occlusion of the proximal MCA, and that 2 h of temporary MCAO with an aneurysm clip results in restoration of blood flow distal to the occlusion. This is consistent with the PbtO_2_ data, which suggests that regional CBF is restored after temporary occlusion but completely interrupted after permanent MCAO. This also suggests that a temporary aneurysm clip does not injure the proximal MCA or its endothelium sufficient to prevent reperfusion. Secondly, the patterns of diffusion deficits in both MCAO groups at 4 h were similar to the size of infarct on H&E at the same time point (Figures 5 and 8). Diffusion weighted imaging remains highly sensitive and specific in early clinical detection of acute ischemia [45], and our DWI results improve the translational strength of this model. Finally, lack of early T2 signal change or evidence of mass effect is also consistent with human stroke, in which cerebral edema and raised ICP can take several days to develop [41].

### Study limitations and future directions

Animals were only monitored for 4 h after vessel occlusion or sham to demonstrate histological evidence of cerebral ischemia and prove the feasibility of the surgical approach and different methods of vessel occlusion. However, 4 h is insufficient to comment on final infarct volume or early mortality rates, nor is neurological outcome assessable in a non-survival study. The area of ischemia at 4 h after permanent MCAO may be predictive of final stroke volume, however larger infarcts could potentially develop with longer survival, especially with temporary MCAO and reperfusion in which cell death can be delayed by days, or with the development of secondary injury phenomena such as raised ICP after large strokes. Therefore, the model in its current setup provides no measureable outcomes for which to test novel therapeutic agents besides early histological changes, MRI diffusion or brain tissue oxygen partial pressures.

In regards to approaching the sheep MCA, the surgery itself is technically demanding when compared with intraluminal rodent models. We found that a thorough knowledge of the relevant anatomy is paramount in safely and efficiently performing the surgery. Another disadvantage of the approach is the amount of extracranial bone and muscle dissection required, which may be a source of not insignificant morbidity for the sheep and may not in its present form be necessarily suitable for a survival study. A more rostral craniotomy that avoids removal of the coronoid process limits the amount of soft tissue and bone dissection and has already been used in a survival study [12], however this approach only provides access to the distal cortical MCA branches, not the important pre-bifurcation proximal segment and associated deep perforating branches.

It would be difficult to achieve the 28% stroke area we observed after permanent MCAO when targeting only the distal vasculature; performing a proximal occlusion and producing complete MCA territory stroke is an important model to simulate the human condition. Malignant MCA stroke is typically associated with larger infarct volumes [46], [47], so for an animal model to replicate malignant MCA stroke and its associated comorbidities, including cerebral edema, raised ICP and high early mortality, occluding the MCA proximally and probably permanently is critically important. Alternatively, smaller cortical-based infarcts sparing the subcortical structures could be performed by transient proximal MCAO followed by reperfusion or by the distal MCAO method as described by Boltze et al. [12], with each approach having its advantages and disadvantages.

## Conclusions

We have produced a large animal model of proximal MCA vessel occlusion in the sheep whereby temporary or permanent vessel occlusion is possible. This model may represent a supplement to the currently used small animal models to improve the translation from experimental studies to clinical therapies.

## Supporting Information

Video S1 **Surgical approach to the proximal Middle Cerebral Artery, demonstrating placement of a straight mini aneurysm clip to produce temporary arterial occlusion.**
(WMV)
